# Prevalence of Gestational Diabetes Mellitus and Its Associated Risk Factors Among Pregnant Women Attending a Tertiary Care Hospital in Bangladesh

**DOI:** 10.1002/edm2.70268

**Published:** 2026-06-22

**Authors:** Mohammad Moin Shahid, Khadija Begum, Kaniz Rahman, Kaniz Fatema, Fatema Tuj Johora

**Affiliations:** ^1^ Department of Endocrinology Ad‐Din Women's Medical College Dhaka Bangladesh; ^2^ Department of Internal Medicine Ad‐Din Women's Medical College Dhaka Bangladesh; ^3^ Department of Dermatology Ad‐Din Women's Medical College Dhaka Bangladesh; ^4^ Ad‐Din Women's Medical College Dhaka Bangladesh

## Abstract

**Background:**

Gestational diabetes mellitus (GDM) is an increasingly important public health problem associated with adverse maternal and fetal outcomes. The burden of GDM is rising in South Asia, including Bangladesh, yet data from tertiary care settings remain limited.

**Objective:**

To determine the prevalence of gestational diabetes mellitus and identify its associated risk factors among pregnant women attending a tertiary care hospital in Bangladesh.

**Methods:**

This hospital‐based analytical cross‐sectional study was conducted from January to June 2025 among pregnant women attending antenatal clinics at a tertiary care hospital in Bangladesh. A consecutive sampling technique was used, and 650 eligible pregnant women undergoing oral glucose tolerance testing (OGTT) were enrolled. GDM was diagnosed according to the International Association of Diabetes and Pregnancy Study Groups (IADPSG) criteria. Sociodemographic, anthropometric, clinical, and obstetric variables were collected using structured interviews and medical records. Independent‐samples t‐tests, chi‐square tests, and multivariable logistic regression analyses were performed using SPSS version 23. Adjusted odds ratios (AORs) with 95% confidence intervals (CIs) were calculated. Women with GDM were significantly older and had higher obesity prevalence and BMI compared with non‐GDM women. Family history of diabetes, polycystic ovary syndrome (PCOS), previous delivery of a baby weighing > 3.5 kg, and history of eclampsia were significantly associated with GDM. Multivariable logistic regression identified increasing maternal age, family history of diabetes, and PCOS as independent predictors of GDM.

**Conclusion:**

GDM is highly prevalent among pregnant women attending tertiary care facilities in Bangladesh. Advanced maternal age, positive family history of diabetes, and PCOS are important independent predictors. Early risk‐based screening and preventive interventions may help reduce maternal and fetal complications associated with GDM.

## Graphical abstract

The graphical abstract summarizes the study design, prevalence, and major risk factors of gestational diabetes mellitus among Bangladeshi pregnant women. It highlights the 32.34% prevalence of GDM, key independent predictors identified by multivariable logistic regression, and emphasizes the importance of early screening, risk stratification, and preventive antenatal interventions.

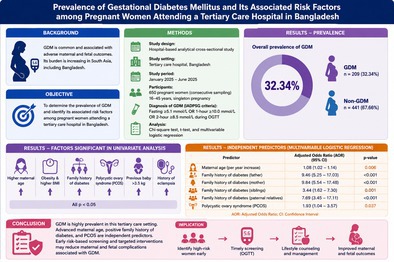

## 1 Introduction

Gestational diabetes mellitus (GDM) is a condition characterized by glucose intolerance first recognized during pregnancy. It presents significant health challenges for both mother and offspring, including increased risks of hypertensive disorders, fetal macrosomia, neonatal hypoglycemia, and future type 2 diabetes mellitus in both mother and child. Understanding the prevalence and determinants of GDM in Bangladesh—particularly within tertiary care settings—is crucial for effective maternal health planning and intervention.

Globally, the prevalence of GDM varies substantially due to differences in diagnostic criteria, screening practices, and population characteristics. The International Association of Diabetes and Pregnancy Study Groups (IADPSG) criteria estimate a global GDM prevalence of about 14.7%, with higher figures reported in South Asia [1].

In Bangladesh, available research indicates that the prevalence of GDM in hospital and population settings ranges widely, often influenced by diagnostic standards and local demographic factors.

In a tertiary care hospital setting, the reported prevalence of GDM has varied:

- Multiple cross‐sectional studies at tertiary hospitals in Dhaka reported a GDM prevalence of > 35% among pregnant women. This relatively high figure may relate to hospital‐based case mix and diagnostic approaches [2].
- Population‐based data from the Bangladesh Demographic and Health Survey 2017–2018 showed an overall national GDM prevalence of about 35%, indicating a substantial burden among Bangladeshi pregnant women when broader WHO criteria are applied [2, 3].
- Other tertiary or hospital‐based investigations in Bangladesh have reported a broad prevalence range (from 7% to over 40%) depending on diagnostic definitions, sampling, and clinical practices [3].

These studies highlight that GDM is a significant public health issue in tertiary care obstetric populations—often higher than community estimates, likely due to referral patterns and higher‐risk case attendance.

Several maternal risk factors have been consistently associated with increased likelihood of GDM in tertiary care contexts:

Multiple studies show that women aged ≥ 25 years have a significantly higher risk of developing GDM compared to younger women. For example, data from the DHS analysis indicated that women ≥ 25 were approximately twice as likely to have GDM [4, 5, 6].

Urban residence has emerged as a strong correlate of GDM, potentially reflective of lifestyle changes like reduced physical activity and a higher prevalence of overweight/obesity among urban women [7].

High pre‐pregnancy BMI and excessive weight gain in pregnancy are common predictors of GDM. Elevated BMI reflects underlying insulin resistance and metabolic strain that contribute to glucose intolerance [8].

A positive family history of type 2 diabetes is repeatedly shown to increase the risk of GDM, consistent with genetic and shared lifestyle risk pathways [9].

Multiparity and a history of macrosomia (large babies) or adverse pregnancy outcomes have been linked to higher GDM risk in tertiary care samples, suggesting that past reproductive history influences glucose metabolism during subsequent pregnancies [8, 10, 11].

The study primarily aims to quantify the burden of gestational diabetes mellitus (GDM) among pregnant women attending a tertiary care hospital in Bangladesh, given the wide variation in reported prevalence across different settings. Additionally, it seeks to systematically identify and evaluate key maternal risk factors for GDM in this population. These include sociodemographic characteristics, anthropometric measures such as body mass index, clinical history, family history of diabetes, and previous obstetric outcomes.

By examining these factors together, the study intends to clarify which variables are most strongly linked to GDM in a tertiary care context, thereby contributing to improved risk stratification, early screening strategies, and targeted preventive interventions for maternal and fetal health in Bangladesh.

## 2 Methods

This hospital‐based analytical cross‐sectional study was conducted in the Department of Obstetrics and Gynaecology of a tertiary care hospital in Bangladesh from January 2025 to June 2025. Pregnant women attending antenatal visits were approached. Bangladeshi women aged 16–45, with a singleton pregnancy, were considered for the study. A consecutive sampling technique was used, where all eligible pregnant women attending the antenatal clinic during the study period were enrolled sequentially until the required sample size was achieved. Pregnant women with pre‐existing diabetes, newly diagnosed overt diabetes during pregnancy, chronic steroid use, severe hyperemesis gravidarum, and acute illness were excluded from the study.

The standard formula is as follows:

$$n=Z2p\left(1-p\right)/d2$$

where *n* = required sample size; *Z* = *Z* value at 95% confidence interval = 1.96; *p* = expected prevalence of GDM = 35% = 0.35; *d* = margin of error = 5% = 0.05.

Substituting the values:

$$n=\left(1.96\right)\;2\times 0.35\times \left(1-0.35\right)/\left(0.05\right)\;2$$

$$n=\left(3.8416\times 0.35\times 0.65\right)/\left(0.0025\right)$$

n=0.873964/0.0025

n≈349.6

Therefore, the minimum required sample size was approximately 350 participants.

If a 10% non‐response or incomplete data rate is added:

$$350+\left(350\times 0.10\right)=385$$

So, the adjusted minimum sample size becomes approximately 385 participants.

Of the 1200 approached cases, 650 pregnant women who met the eligibility criteria and underwent the Oral Glucose Tolerance Test (OGTT) during the study period were enrolled. GDM was diagnosed if any of the following plasma glucose values during OGTT were met or exceeded: fasting plasma glucose ≥ 5.1 mmol/L, 1‐h plasma glucose ≥ 10.0 mmol/L, or 2‐h plasma glucose ≥ 8.5 mmol/L (IADPSG criteria).

According to the IADPSG, hyperglycemia first detected during pregnancy is categorized into two groups:

1. Gestational Diabetes Mellitus (GDM), and
2. Diabetes Mellitus in Pregnancy (DIP), also referred to as overt diabetes in pregnancy.

In the present study, women with previously diagnosed diabetes mellitus and newly diagnosed overt diabetes during pregnancy were excluded from enrollment. Therefore, all abnormal glucose tolerance cases identified during screening were classified as GDM, and no separate DIP subgroup emerged in the analysis.

Participants were categorized into two groups based on OGTT results: gestational diabetes mellitus (GDM) and non‐GDM.

Sociodemographic, clinical, anthropometric, and obstetric information were collected using a structured data sheet and patient interview. Variables included maternal age, residence (urban/sub‐urban/rural), gravidity, history of polycystic ovary syndrome (PCOS), obesity status, body mass index (BMI), family history of diabetes (father, mother, sibling, paternal and maternal family), prior obstetric events (abortion, premature rupture of membrane‐PROM, macrosomia, baby weight > 3.5 kg, eclampsia), thyroid disorders, and hypertension.

Height and weight were measured using standard procedures, and BMI was calculated as kg/m^2^. Obesity was defined using standard BMI cutoffs.

Data were analysed using SPSS23, IBM, United States of America. Continuous variables were expressed as mean ± standard deviation (SD) and compared using independent‐samples ‘*t*‐tests’. Categorical variables were presented as percentages and compared using chi‐square tests. Multivariable logistic regression analysis was performed to identify independent predictors of GDM. Variables found clinically relevant or statistically significant in univariate analysis were entered into the regression model. Adjusted odds ratios (AORs) with 95% confidence intervals (CIs) were calculated. A *p*‐value < 0.05 was considered statistically significant. The prevalence of GDM was calculated as the proportion of diagnosed cases among the total tested population. Maternal age, BMI, obesity, family history variables, PCOS, previous baby > 3.5 kg, and eclampsia were entered into the multivariable logistic regression model.

Ethical approval for the study was obtained from the Institutional Review Board of the respective institution before data collection. Written informed consent was obtained from all participants before enrollment. Confidentiality and anonymity of participant information were strictly maintained throughout the study.

## 3 Results

The overall prevalence of gestational diabetes mellitus was 32.34% among pregnant women who underwent OGTT at the tertiary care hospital.

### 3.1 Maternal Characteristics (Table 1)

**TABLE 1:** Characteristics of the cases.

Particulars	GDM	Non‐GDM	*p*
Age (Mean ± SD)	28.46, 4.29	27.12, 4.36	0.001
Address			
Urban	62.57%	56.43%	0.245
Sub‐Urban	33.12%	35.96%	
Rural	4.29%	7.60%	
Family history of diabetes (father)	37.42%	16.37%	0.001
Family history of diabetes (mother)	43.55%	17.83%	0.001
Family history of diabetes (sibling)	15.95%	6.14%	0.001
Family history of DM (paternal family)	14.72%	5.55%	0.001
Family history of DM (maternal family)	8.58%	6.72%	0.452
Obesity	69.32%	54.67%	0.005
History of PCOS	20.24%	11.40%	0.008
History of abortion	16.56%	25.43%	0.026
History of PROM	4.90%	2.63%	0.185
History of macrosomia	1.22%	0.87%	0.710
History of baby weight > 3.5 kg	12.26%	4.97%	0.003
History of eclampsia	5.52%	1.16%	0.004
History of hypothyroidism	7.97%	15.78%	0.017
History of hyperthyroidism	0.611%	0	0.147
History of hypertension	1.22%	0	0.040
Primi	29.44%	32.74%	0.456
Multigravida	69.93%	64.03%	0.309
Gestational week while performing OGTT (Mean SD)	22.568.55	20.668.22	0.017
BMI (Mean SD)	29.6684.73	28.064.98	0.001

Mean maternal age was significantly higher in the GDM group compared with the non‐GDM group (28.46 ± 4.29 vs. 27.12 ± 4.36 years, *p* = 0.001). Distribution of residence (urban, suburban, rural) did not differ significantly between groups (*p* = 0.245).

Women with GDM showed greater adiposity overall, demonstrated by both higher obesity prevalence (69.32% vs. 54.67%, *p* = 0.005) and elevated mean BMI values (29.67 ± 4.73 vs. 28.06 ± 4.98 kg/m^2^, *p* = 0.001).

### 3.2 Family History of Diabetes (Table 1)

A strong association was observed between GDM and family history of diabetes. History of diabetes in father (37.42% vs. 16.37%), mother (43.55% vs. 17.83%), sibling (15.95% vs. 6.14%), and paternal family (14.72% vs. 5.55%) was all significantly higher in the GDM group (all *p* = 0.001). Maternal family history was not significantly different (*p* = 0.452).

### 3.3 Reproductive and Medical Risk Factors (Table 1)

The history of PCOS was more frequent among GDM women (20.24% vs. 11.40%, *p* = 0.008). Previous abortion was less common in the GDM group (16.56% vs. 25.43%, *p* = 0.026).

Prior delivery of a baby weighing greater than 3.5 kg was more frequently observed with GDM (12.26% vs. 4.97%, *p* = 0.003). History of eclampsia (5.52% vs. 1.16%, *p* = 0.004) and hypertension (1.22% vs. 0%, *p* = 0.040) were more prevalent among GDM mothers. The history of PROM and macrosomia did not show a significant association.

Hypothyroidism was more common in the non‐GDM group (15.78% vs. 7.97%, *p* = 0.017), while hyperthyroidism showed no significant difference (*p* = 0.147).

There was no significant difference between groups regarding gravidity status (primi vs. multigravida).

### 3.4 Multivariable Logistic Regression Analysis (Table 2)

**TABLE 2:** Multivariable logistic regression analysis for predictors of GDM.

Variable	Adjusted odds ratio (AOR)	95% CI	*p*
Maternal age	1.08	1.02–1.14	0.006
BMI	1.05	0.99–1.11	0.126
Obesity	1.44	0.82–2.54	0.203
Family history of DM (father)	9.46	5.25–17.03	< 0.001
Family history of DM (mother)	9.84	5.54–17.48	< 0.001
Family history of DM (siblings)	3.44	1.62–7.30	0.001
Family history of DM (paternal family)	7.69	3.45–17.11	< 0.001
PCOS	1.93	1.04–3.57	0.037
Previous baby > 3.5 kg	2.16	0.97–4.81	0.060
Eclampsia	3.90	0.85–17.89	0.080

Multivariable logistic regression analysis was performed to identify independent predictors of gestational diabetes mellitus. Increasing maternal age remained independently associated with GDM (AOR: 1.08, 95% CI: 1.02–1.14, *p* = 0.006). A strong association was observed between GDM and family history of diabetes in father (AOR: 9.46, 95% CI: 5.25–17.03, *p* < 0.001), mother (AOR: 9.84, 95% CI: 5.54–17.48, *p* < 0.001), siblings (AOR: 3.44, 95% CI: 1.62–7.30, *p* = 0.001), and paternal family members (AOR: 7.69, 95% CI: 3.45–17.11, *p* < 0.001).

The history of polycystic ovary syndrome (PCOS) also emerged as an independent predictor of GDM (AOR: 1.93, 95% CI: 1.04–3.57, *p* = 0.037). Although higher BMI, obesity, prior delivery of a baby weighing > 3.5 kg, and history of eclampsia showed positive associations, these variables did not retain statistical significance after adjustment for confounding factors.

### 3.5 Timing of Testing and Glycemic Values (Table 3)

**TABLE 3:** Values of oral glucose tolerance test (OGTT).

	GDM	Non‐GDM	*p*
FBS (mmol/L)	5.7931.43	4.480.34	0.001
1‐h glucose (mmol/L)	11.432.05	8.211.17	0.001
2‐h glucose (mmol/L)	9.6992.14	6.761.11	0.001

Mean gestational week at OGTT testing was higher in the GDM group (22.56 ± 8.55 vs. 20.66 ± 8.22 weeks, *p* = 0.017).

All glucose parameters were significantly elevated among GDM women:

- Fasting glucose: 5.79 ± 1.43 versus 4.48 ± 0.34 (*p* = 0.001)
- 1‐h glucose: 11.43 ± 2.05 versus 8.21 ± 1.17 (*p* = 0.001)
- 2‐h glucose: 9.70 ± 2.14 versus 6.76 ± 1.11 (*p* = 0.001)

## 4 Discussion

**FIGURE 1:**
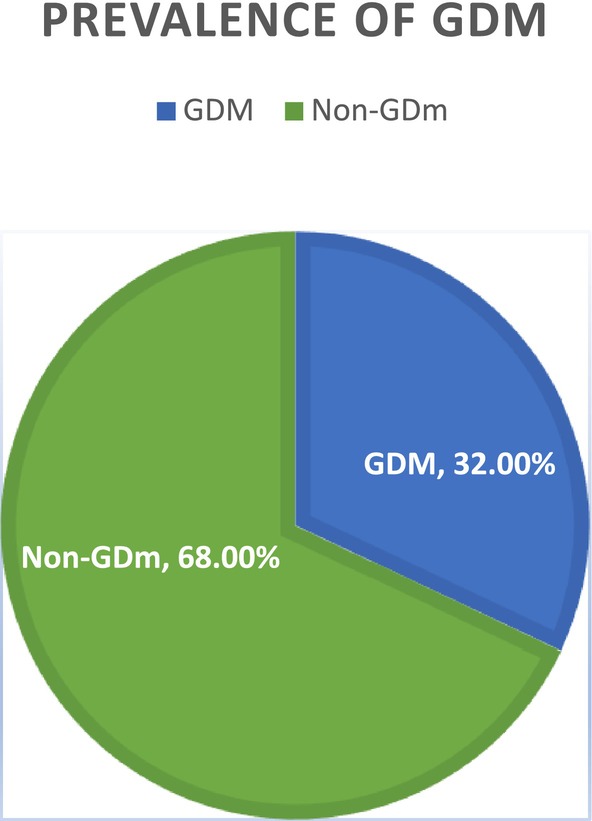
Prevalence of GDM.

This hospital‐based analytical cross‐sectional study demonstrated a high prevalence of gestational diabetes mellitus (GDM) (32.34%) among pregnant women attending a tertiary care hospital in Bangladesh (Figure 1). The observed prevalence is comparable to findings from other tertiary and urban South Asian settings, where broader screening strategies and referral of high‐risk pregnancies contribute to higher detection rates [1, 12].

Maternal age was significantly associated with GDM, and multivariable logistic regression analysis confirmed increasing maternal age as an independent predictor of the disease. This finding is consistent with previous studies showing that advancing maternal age is associated with progressive insulin resistance, reduced pancreatic β‐cell compensation, and impaired glucose tolerance during pregnancy. The higher prevalence of GDM among older mothers highlights the importance of age‐based risk stratification during antenatal care [12, 13, 14].

Measures of adiposity, including obesity and elevated BMI, were significantly higher among women with GDM in univariate analysis. These findings support the established role of maternal overweight and obesity in the pathogenesis of GDM through increased insulin resistance and metabolic [12, 15].

However, after adjustment in multivariable analysis, BMI and obesity did not retain independent statistical significance, suggesting that their effects may partly overlap with other metabolic and familial risk factors within this population.

A particularly strong association was observed between GDM and family history of diabetes involving first‐degree relatives and paternal family members. Multivariable logistic regression demonstrated that family history of diabetes remained one of the strongest independent predictors of GDM. This finding emphasizes the combined influence of genetic susceptibility, shared environmental exposures, and familial lifestyle patterns on glucose metabolism during pregnancy. The results are biologically plausible and consistent with previous evidence demonstrating familial clustering of glucose intolerance and type 2 diabetes [12, 15, 16, 17, 18, 19].

The history of polycystic ovary syndrome (PCOS) was also independently associated with GDM. Both conditions share insulin resistance as a central pathophysiological mechanism, which may explain the increased susceptibility of women with PCOS to develop glucose intolerance during pregnancy. In addition, previous delivery of a baby weighing more than 3.5 kg and a history of eclampsia were more common among women with GDM, suggesting that adverse obstetric outcomes may serve as clinical indicators of underlying metabolic dysfunction and future pregnancy‐related glucose intolerance [11, 20, 21].

Interestingly, hypothyroidism was more frequent in the non‐GDM group, which contrasts with several previously published studies reporting positive associations between thyroid dysfunction and GDM [22]. This unexpected finding may reflect differences in treatment status, screening practices, sample composition, or residual confounding factors. The relatively small subgroup size may also have influenced this observation. Further prospective studies are needed to clarify the relationship between thyroid disorders and GDM in Bangladeshi populations.

Residence status and gravidity did not show significant associations with GDM in this study. This may be explained by the mixed referral nature of tertiary care hospitals, where women from diverse geographic and sociodemographic backgrounds receive care, thereby reducing contrasts between groups.

The findings of this study have important clinical and public health implications. Early identification of women at increased risk of GDM, particularly those with advanced maternal age, obesity, PCOS, and a positive family history of diabetes, may improve targeted screening and preventive interventions. Strengthening antenatal screening programs and promoting lifestyle modification strategies could help reduce maternal and fetal complications associated with GDM in Bangladesh.

This study has several strengths, including a relatively large sample size, use of standardized OGTT‐based IADPSG diagnostic criteria, and evaluation of multiple clinical and obstetric risk factors. Nevertheless, several limitations should be acknowledged. The cross‐sectional design limits causal inference between risk factors and GDM. As this was a single‐center tertiary care study, the findings may not be fully generalizable to the broader Bangladeshi population, particularly rural communities. Referral bias may also have contributed to the relatively high prevalence observed because tertiary hospitals commonly manage high‐risk pregnancies. Some variables relied on participant recall and may therefore be subject to recall bias. In addition, lifestyle‐related variables such as dietary habits, physical activity, and socioeconomic status were not evaluated. Despite these limitations, the inclusion of multivariable logistic regression analysis strengthened the identification of independent predictors of GDM.

## 5 Conclusion

Gestational diabetes mellitus is a prevalent condition among pregnant women attending tertiary care hospitals in Bangladesh. Increasing maternal age, family history of diabetes, and polycystic ovary syndrome were identified as independent predictors of GDM. These findings underscore the need for comprehensive screening programs coupled with targeted health promotion and management strategies in tertiary and primary care settings alike.

## Author Contributions

**Kaniz Fatema:** data curation. **Mohammad Moin Shahid:** conceptualization, data curation, formal analysis, visualization, writing – original draft, writing – review and editing, project administration, supervision, investigation, methodology, software, validation, funding acquisition, resources. **Fatema Tuj Johora:** data curation. **Khadija Begum:** data curation. **Kaniz Rahman:** data curation.

## Conflicts of Interest

The authors declare no conflicts of interest.

## Data Availability

Research data are not shared.
